# Supplementary material for: Identification of photoperiod-induced specific miRNAs in the adrenal glands of Sunite sheep (Ovis aries)
Source: Front Vet Sci. 2022 Jul 22;9:888207. doi: 10.3389/fvets.2022.888207 (PMC9354845; doi:10.3389/fvets.2022.888207)
Supplement: Supplementary file 1 [file Data_Sheet_1.ZIP › Supplementary file/miRDeep2 file.zip!/miRDeep2 file/Novel_Structure/LP42A2/Novel_15.pdf]

Provisional ID	:	NC_019474.2_27964
Score total	:	2.4
Score for star read(s)	:	-1.3
Score for read counts	:	0
Score for mfe	:	2.1
Score for randfold	:	1.6
Score for cons. seed	:	
Total read count	:	62
Mature read count	:	62
Loop read count	:	0
Star read count	:	0

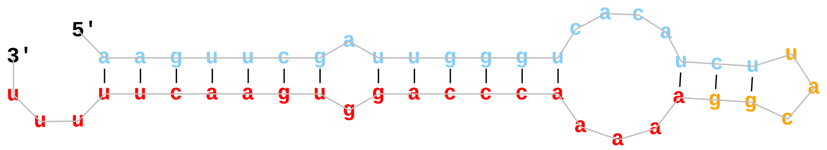

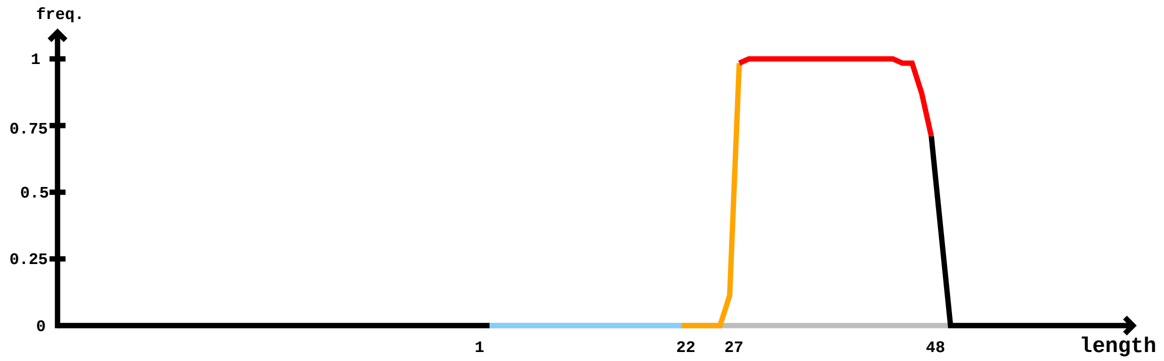

Star **Mature**

```
5'- ccgcguggacuccagccuggggauaacuguuggguuggccaaaaaguucgauugggucacaucuuacggaaaaacccaggugaacuuuuuuggccaacucaacaauacac   -3'   exp
    ....((...(((((...)))))...))(((((((((((((((((((((((.((((((....(((...)))...)))))).))))).))))))))))))))))))......   reads   mm   sample
    ....................................................................gaaaaacccagAugaacuuuu.....................   6       1    seq
    ....................................................................gaaaaacccagAugaacuuuuu....................   1       1    seq
    .....................................................................aaaaacccagAugaacu........................   1       1    seq
    .....................................................................aaaaacccagAugaacuuu......................   7       1    seq
    .....................................................................aaaaacccagAugaacuuuu.....................   4       1    seq
    .....................................................................aaaaacccagAugaacuuuuu....................   42      1    seq
    ......................................................................aaaacccaggugaacuuuuu....................   1       0    seq
```
